# Methodologies for Assessing Chemical Toxicity to Aquatic Microorganisms: A Comparative Review

**DOI:** 10.3390/molecules31030485

**Published:** 2026-01-30

**Authors:** Hong Chen, Yao Li, Quanzhan Chen, Changyun Chen, Yaojuan Hu

**Affiliations:** Jiangsu Key Laboratory of Zero-Carbon Energy Development and System Integration, School of Environmental Science, Nanjing Xiaozhuang University, Nanjing 211171, China

**Keywords:** aquatic microorganisms, toxicity assessment, spectroscopies, mass spectrometry, nanobiochips

## Abstract

Aquatic ecological issues have garnered significant attention in recent years, driving the demand for convenient, effective, and systematic assessment methods in environmental risk evaluation. This review provides a comprehensive introduction to methodologies for assessing the toxicity of chemicals toward aquatic microorganisms, which include viruses, bacteria, fungi, protozoa, and algae. Among these, microalgae are commonly used as model organisms due to their relative simplicity. The article details conventional biological methods, general chemical techniques, modern instrumental analyses, and informatics approaches, with a particular focus on algae and bacteria as model organisms for toxicity assessment. The principles, advantages, and limitations of each method are discussed, along with examples of their application in various contexts. Biological methods offer direct visualization, convenience, and rapid results, while modern instrumental techniques enable mechanistic insights at molecular and biochemical levels. Informatics methods facilitate toxicity evaluation in complex systems. While aquatic microorganisms encompass viruses, fungi, protozoa, bacteria, and algae, this review primarily focuses on bacteria and algae as model organisms due to their ecological relevance, sensitivity, and widespread use in standardized assays.

## 1. Introduction

With the increasing complexity of industrial activities, pollution and damage to the ecological environment have become increasingly severe. The large-scale discharge of various pollutants significantly impacts living environments; however, the distribution and toxic mechanisms of these pollutants remain inadequately understood, hampering effective pollution control and prevention. As a vital ecological system essential for survival and development, the aquatic environment is particularly vulnerable. However, accidents of water pollution occur frequently, such as natural disasters, chemical production, transportation, storage, use, and subsequent discharge. Toxicity refers to the capacity of a substance to induce cellular dysfunction upon contact with or entry into a susceptible part of an organism [1,2]. Highly toxic substances can cause damage even at very low doses or concentrations. The accumulation of toxic pollutants in water bodies adversely affects aquatic ecosystems, with some substances exhibiting potent toxicity at trace levels. Therefore, developing multi-parameter and accurate toxicity testing methods is crucial for elucidating the toxic effects of pollutants on aquatic microorganisms and for assessing the environmental risks of chemicals [1]. In recent years, a panel of toxicity testing methods based on aquatic microorganisms have been established and are rapidly advancing [2,3].

Aquatic microorganisms encompass bacteria, viruses, fungi, protozoa, and microalgae, with predominant research focusing on bacteria and algae [4]. As prokaryotes, bacteria possess simple structures and rapid reproduction rates, playing essential roles in natural nutrient cycling. Their responses to toxins, such as enzyme inhibition [5,6], membrane disruption [7], and changes in physiological behavior [8], serve as effective indicators of water pollution. As primary producers, algae absorb inorganic nutrients and CO_2_ through photosynthesis, forming the basis of energy flow and substance transfer in aquatic ecosystems. As primary responders to pollution, algae are highly sensitive bioindicators for assessing aquatic toxicity [9]. They are often more susceptible to toxic substances than fish or crustaceans. Their rapid life cycles and ease of cultivation also facilitate efficient toxicity testing and enable direct cellular-level observation.

This review was conducted based on a systematic search of the scientific literature up to 2026. Primary databases included Web of Science, Scopus, and PubMed. Key search terms encompassed ‘aquatic microorganism toxicity assessment’, ‘bioassay’, ‘(ecot)oxicology’, ‘spectroscopy (FTIR, hyperspectral)’, ‘mass spectrometry (ICP-MS, GC-MS, LC-MS)’, ‘nanobiochip’, ‘QSAR’, and ‘machine learning’ in various combinations. The focus was placed on selecting seminal and high-impact studies that illustrate methodological principles and applications, with a particular emphasis on algae and bacteria as the most prevalent model organisms.

**Figure 1:**
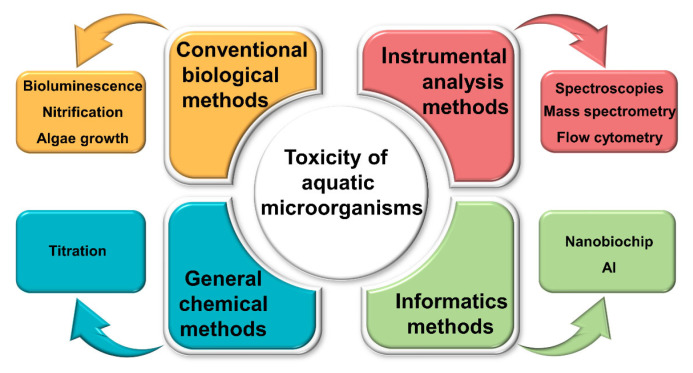
Current methodological framework for toxicity assessment of aquatic microorganism.

This review aims to provide a critical synthesis and comparative framework for these methods, distinguishing itself through an integrated analysis of classical and emerging approaches—including computational informatics as part of the technology landscape—and by offering practical selection criteria based on performance parameters such as sensitivity, cost, and throughput. This review systematically synthesizes classical and emerging methodologies, with a focus on bacteria and algae as representative models, to provide a comparative framework for researchers and practitioners. We also highlight integrative approaches combining experimental and computational tools for enhanced toxicity prediction and mechanistic understanding. A schematic overview of the current methodological framework is provided in Figure 1. It covers four main categories: conventional biological assays, general chemical methods, modern instrumental techniques, and informatics-based approaches. Specific technologies include routine toxicity tests, spectroscopy, mass spectrometry, nanobiochips, and related tools. The principles, advantages, and limitations of each method are discussed in detail.

## 2. General Toxicity Assays

General methods for assessing toxicity in aquatic microorganisms, including biological and chemical assays, require minimal specialized equipment and primarily assess acute toxicity. Key approaches include luminescent-bioluminescence inhibition tests, nitrification inhibition tests, and algae growth inhibition tests.

### 2.1. Biological Method

Bioassays measure the effects of pollutants or environmental factors by monitoring biological endpoints (e.g., mortality or enzyme inhibition) [10]. Their advantages include cost-effectiveness, operational ease, direct toxicity assessment, and the ability to detect interactions between pollutants (e.g., synergism or antagonism). The tests provide ecologically relevant data for establishing safe contaminant thresholds and supporting wastewater regulations and ecological risk frameworks [11]. Based on duration, bioassays are classified as acute (short-term, rapid, and economical) [11], chronic (long-term, life-cycle studies determining MATC (maximum acceptable toxicant concentration)) [12], or subchronic (intermediate duration) [13]. Standardized acute toxicity methods from organizations such as ISO, USEPA, and OECD are widely used. An overview of common standardized bioassays is presented in Table 1, and three common examples are described below.

#### 2.1.1. Luminescent Bacteria-Bioluminescence Inhibition Test

This toxicity assay employs luminescent bacteria (e.g., *Vibrio*, *Luminococcus*), which are Gram-negative facultative anaerobes [20]. The test quantifies toxicity by measuring the decline in bioluminescence resulting from the inhibition of luciferase or cellular respiration by pollutants [21]. It is a rapid, low-cost method highly suitable for detecting heavy metal toxicity [22].

Research employing this method varies in its specific focus and application contexts, which can be categorized as follows:

- Ecological Impact Assessment with Standardized Systems: Commercial, standardized bioassays such as Microtox® (which utilizes V. fischeri) have been applied to evaluate the ecological impact of pollutants in complex environmental matrices. For instance, this approach has been used to assess heavy metal bioavailability in estuarine sediments and their resultant ecological impacts [23].
- Site-Specific Environmental Monitoring: Kinetic bioluminescence inhibition tests with V. fischeri have been deployed for site-specific pollution monitoring. An example includes its application in assessing toxicity in sediments from mining-impacted estuaries in northern Spain, providing direct evidence of localized environmental stress [24].
- Comparative Toxicity Profiling under Controlled Conditions: Studies utilizing V. fischeri in controlled media conditions (e.g., saline versus buffered) aim at fundamental comparative toxicity profiling. This approach has been effectively used to generate comparative toxicity data for specific pollutant classes, such as rare earth elements and their oxides [25].
- Methodological Expansions and Applications: Beyond these V. fischeri-centric applications, methodological advancements have expanded the technique’s utility. Enhanced thermal stability, crucial for field applications in varied climates, has been achieved by employing alternative strains such as *Photobacterium* sp. MIE [21]. The approach is also widely used in pesticide risk assessment, as pesticides frequently enter aquatic systems and affect microorganisms [26]. Suspect screening studies highlight pesticides as key contaminants of concern in surface waters [26]. The method enables comparative toxicity screening of pesticides and solvents [27], analysis of non-additive mixture effects, and the development of portable field detectors. For example, the Biotox™ test has been used to determine EC_50_ values for various pesticides, identifying substances like pentachlorophenol as highly toxic and demonstrating complex mixture interactions [27]. Portable field instruments utilizing optimized strains such as *Vibrio rosenbergii* and standardized reagents have been developed for rapid on-site measurement, allowing parallel sample processing under less stringent temperature control while maintaining consistency with ISO 9509 procedures [28].

#### 2.1.2. Nitrification Inhibition Test (ISO 9509)

This ISO-standardized method evaluates toxicity by measuring the inhibition of nitrifying bacteria. The nitrification process consists of two steps: ammonia oxidation to nitrite by ammonia-oxidizing bacteria (AOB), and nitrite oxidation to nitrate by nitrite-oxidizing bacteria (NOB). Due to their slow growth and high sensitivity, AOB are particularly susceptible to inhibition, making the first step a critical indicator [29]. Tests may employ pure strains (e.g., *Nitrosomonas*, *Nitrobacter*) or mixed cultures from activated sludge. Although less prevalent than luminescent bacteria assays, this method has been effectively applied in specific scenarios. For example, it has been used to associate nanosilver toxicity with ROS generation and disrupted oxygen uptake in nitrifying bacteria [30], and to demonstrate the heightened sensitivity of chlorinated phenols in nitrification inhibition compared to general bacterial toxicity tests [31].

#### 2.1.3. Algae Growth Inhibition Assessment

Algae are highly sensitive bioindicators of water pollution, with toxicity responses observed in growth, reproduction, morphology, photosynthesis, and enzymatic activity [32,33]. Commonly used test species such as diatoms, *Scenedesmus*, and *Chlorella* are applied to assess the toxicity of heavy metals, nanomaterials, and ionic liquids. For example, *Tisochrysis lutea* has shown equal or greater sensitivity than standard species to various pollutants, including zinc and triclosan [34]. Studies have documented inhibited acetylcholinesterase and oxidative stress in *Moina macrocopa* exposed to gold nanostructures [35], toxic effects of imidazole ionic liquids on *Scenedesmus quadricauda* and *Chlamydomonas reinhardtii* [36], and pharmaceutical impacts on algae [37]. Additionally, copper was found to be 42 times more toxic than cadmium to *Chlamydomonas* sp. using chlorophyll fluorescence-based growth assays [38].

## 3. Instrumental Analytical Methods I: Spectroscopic Techniques

While general bioassays effectively signal the presence of toxicity, they often fall short of revealing its molecular basis. Spectroscopic techniques bridge this gap by providing a non-destructive window into the biochemical alterations induced by pollutants. The following section details how methods such as FTIR, hyperspectral imaging, and fluorescence spectroscopy are employed to link toxic effects to specific molecular changes in aquatic microorganisms.

Spectroscopic techniques probe material properties based on their spectral characteristics, offering advantages such as operational simplicity, non-destructive nature, minimal to no sample preparation, and rapid analysis. Furthermore, integration with advanced technologies enhances their speed and detection accuracy [39,40]. Selected representative techniques and their applications are summarized below. Historically, conventional chemical methods such as titration were used for pollutant quantification, but their limited accuracy and mechanistic insight have led to their replacement by modern instrumental techniques, which are discussed in the following sections.

### 3.1. FTIR

**Figure 2:**
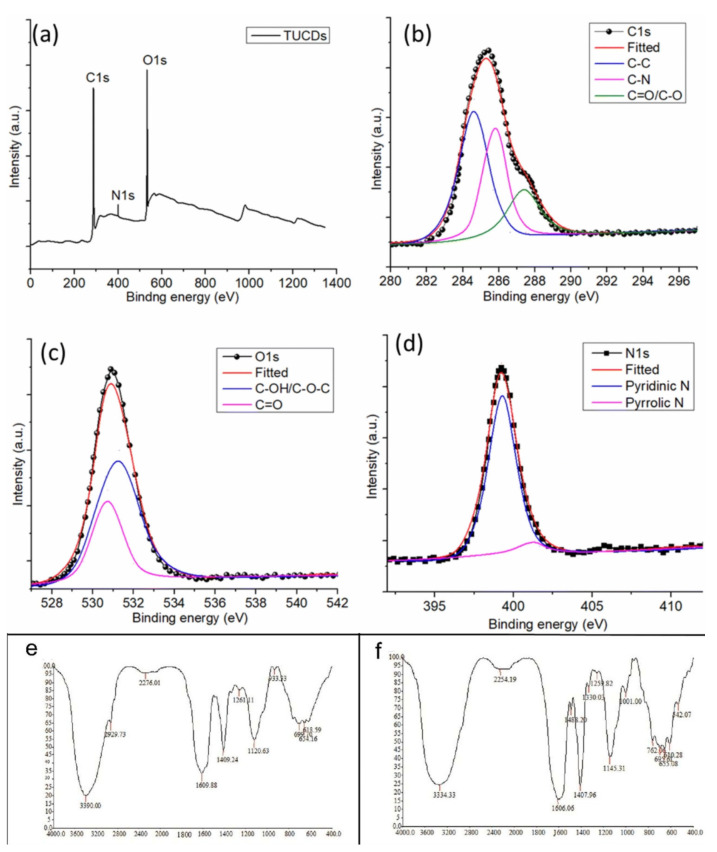
XPS analysis of tamarind-urea derived carbon dots (TUCDs) [43]. (**a**) Wide-scan survey spectrum showing the presence of C, O, and N. High-resolution spectra of (**b**) C 1s, (**c**) O 1s, and (**d**) N 1s. The deconvoluted C 1s peaks correspond to C-C/C=C, C-N/C-O, and C=O bonds, confirming N-doping and oxygen-containing groups. The O 1s and N 1s spectra further detail the chemical states of surface oxygen and the incorporation of nitrogen in various configurations, collectively verifying the heteroatom-doped structure and surface chemistry of the TUCDs. Copyright 2025 Rsc Advances. Fourier Transform Infrared spectrum of control (**e**) and nanoparticle-associated (**f**) cells of C. vulgaris [50]. Copyright 2019 Aquatic Toxicology.

Fourier-transform infrared spectroscopy (FTIR) analyzes molecular vibrations and rotations to generate spectral data for identifying and quantifying compounds [41,42]. As a non-destructive technique, it provides detailed molecular composition information and is widely applied in organic analysis, structural studies, and biomolecular research [43,44]. Its applications extend to diverse fields such as pharmaceuticals, agriculture, food science, environmental monitoring, and toxicology [45,46,47]. FTIR can detect biochemical changes in cells under environmental stress, such as compositional and structural alterations, and, when combined with multivariate analysis, offers sensitive toxicity evaluation and mechanistic insight [48]. For example, Tabish et al. [49] employed FTIR in conjunction with X-ray photoelectron spectroscopy (XPS) to systematically characterize nitrogen-doped carbon dots (CDs) synthesized from tamarind and urea. The FTIR analysis confirmed the presence of characteristic functional groups such as C=O and O-H on the CD surface. Comprehensive XPS analysis provided deeper elemental and chemical state information, as detailed in Figure 2. The survey spectrum (Figure 2a) confirms the co-existence of carbon (C), nitrogen (N), and oxygen (O). High-resolution spectra of C 1s (Figure 2b), O 1s (Figure 2c), and N 1s (Figure 2d) reveal fine chemical structures, with the C 1s spectrum deconvoluted into peaks corresponding to C-C, C-N, and C=O bonds. These spectroscopic results jointly verify successful nitrogen doping and specific surface chemistry. Such multi-technique characterization clarifies the structure–property relationship, providing a molecular-level explanation for the material’s subsequent excellent antioxidant activity (free radical scavenging efficiency >80%) and its high, pH-dependent sensitivity in detecting the oxidative stress metabolite 3-nitrotyrosine, which is associated with neurodegenerative diseases. This study exemplifies the critical value of combining FTIR with XPS in elucidating nanomaterial structure and advancing their applications in biomedical sensing and oxidative stress monitoring.

Xin et al. [51] employed synchrotron-enhanced FTIR to investigate triclosan toxicity under varying environmental conditions, identifying significant interactions among phosphorus, pH, temperature, and salinity. Similarly, Suman et al. [50] used FTIR to detect membrane damage in Chlorella vulgaris caused by ZnO nanoparticles through analysis of functional group modifications.

### 3.2. Hyperspectral Imaging

**Figure 3:**
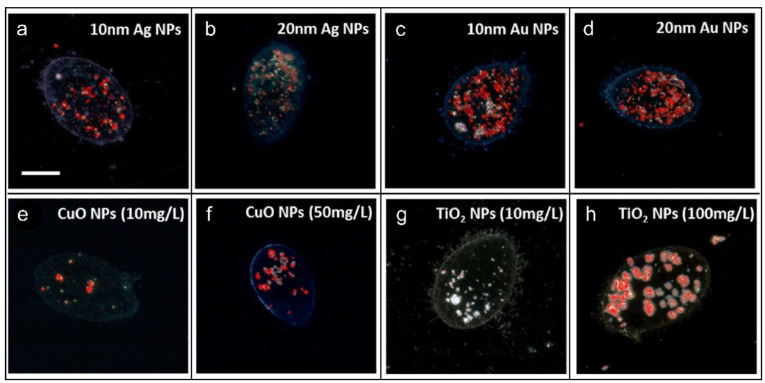
Demonstrative hyperspectral pictures of T. thermophila exposed to NPs for 2 h. (**a**–**d**) Protozoa hatched with 10 nm Ag NPs, 20 nm Ag NPs, 10 nm Au NPs, and 20 nm Au NPs, individually, at 10 mg/L. (**e**–**h**) Protozoa were exposed to 10 and 50 mg/L CuO NPs and 10 and 100 mg/L TiO_2_ NPs, individually. Red dots on the darkfield pictures indicate SAM-localized spectral profiles of the respective NPs. All pictures were captured employing a 100× objective, with the scale bar = 10 μm [57]. Copyright 2014 American Chemical Society.

Hyperspectral imaging (HSI) integrates spectral and spatial imaging to simultaneously capture high-resolution spectral data for each pixel along with visual information, thereby correlating a sample’s morphology with its chemical composition [52,53]. This technique produces geospatially resolved spectral profiles with high resolution across multiple bands, enhancing quantitative analysis by leveraging the inherent spectral properties of materials [54]. Operating rapidly and without direct contact, HSI supports large-scale, real-time monitoring and is especially suited for non-destructive, online tracking of dynamic biological processes such as microalgae growth [55,56]. For example, Mortimer et al. [57] combined HSI with enhanced dark-field microscopy (HSI-M) to detect and monitor Tetrahymena thermophila, a model ciliate in nanotoxicology. By using a spectral library built from nanoparticles (NPs) in extracellular secretions, the system enabled NP detection and semi-quantification. HSI-M distinguished NP types based on spectral profiles shaped by size, aggregation state, and subcellular distribution. Figure 3 confirms intracellular NP presence via localized spectral signals after 2 h exposure. Coupling HSI-M with bioassays (e.g., toxicity or oxidative stress assays) facilitates mechanistic insights into NP toxicity through visualization of NP localization, aggregation behavior, and interactions with extracellular substances.

Schwab et al. [58] assessed multi-walled carbon nanotubes (CNTs) under multiple conditions—including industrial, purified, virgin, oxidized, and soot-containing forms—and their effect on diuron toxicity toward *Chlorella* photosynthesis. Light microscopy and hyperspectral imaging (HSI) revealed no spectral evidence of CNT uptake by algal cells. Instead, diuron toxicity was enhanced due to elevated local exposure concentrations near CNT-associated cells. Angel et al. [59] employed high-resolution imaging and spectroscopy to examine nano-CeO_2_ toxicity in *Chlamydomonas reinhardtii*. Silver tracking after exposure to PVP-coated AgNPs revealed that intracellular accumulation originated from Ag^+^ release and chelation, with subsequent internalization of AgNPs. Fakhrullin et al. [60] reviewed dark-field microscopy (DFM) and hyperspectral enhancements, emphasizing its capacity for label-free nanoscale imaging (≥5 nm) of plasmonic nanoparticles in complex environmental and biological samples such as wastewater and live microorganisms.

### 3.3. Fluorescence

Fluorescence imaging spectroscopy is an emerging technique that associates spatial imaging with spectral analysis [61,62]. It simultaneously captures rich spatial and spectral information from samples. When exposed to ultraviolet light, the test object emits fluorescence at varying intensities. In photosynthetic organisms, chlorophyll fluorescence parameters serve as reliable indicators of photosystem II (PSII) efficiency [63]. Since PSII functions at the onset of photosynthesis, these parameters effectively represent the overall photosynthetic process. A direct impairment of PSII activity, observable through chlorophyll fluorescence, occurs when photosynthesis is disrupted. Analysis of these fluorescence parameters provides a non-invasive and effective means to monitor the photosynthetic process in its entirety. Due to their high sensitivity to toxic pollutants, algal fluorescence parameters are widely used in chlorophyll fluorescence-based assays for assessing toxicity in aquatic microorganisms.

Schreiber et al. [64] developed a novel phytotoxicity bioassay using chlorophyll fluorescence imaging of algal suspensions in multiwell plates. To enhance screening throughput in routine quality control, they designed a dedicated chlorophyll fluorescence imaging system (Maxi-Imaging-PAM) for multi-well plate assays. The model experiment evaluated the herbicide Diuron’s effects on algae, including the diatom *Phaeodactylum tricornutum* and two chlorophytes: *Chlorella vulgaris* (freshwater) and *Desmodesmus subspicatus*. The quantum yield Y(II) was derived from the ratio of two fluorescence values. The formulation is

$$Y(\mathrm{II})=\frac{{F^{\prime }}_{m}-F}{{F^{\prime }}_{m}}$$

(F = Steady-state fluorescence yield under actinic (photosynthetically active) light. $F_{m}^{\prime }$ = Maximum fluorescence yield under light-adapted conditions).

Based on this, the authors propose to quantify phytotoxicity by using the saturation pulse way to evaluate the inhibitory effect of photosynthetic unit II quantum yield Y (II). The formula is

$$\mathrm{Inhibition}(\%)=(1-\frac{{Y(\mathrm{II})}_{\mathrm{sample}}}{{Y(\mathrm{II})}_{\mathrm{control}}})\times 100\%$$

**Figure 4:**
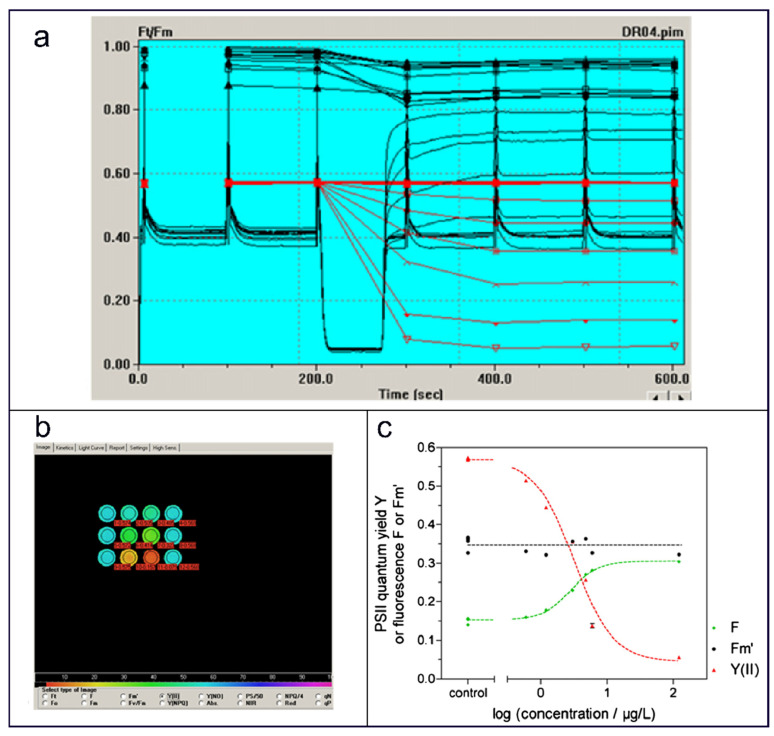
Diuron-induced fluorescence variation in *P. tricornutum* suspensions in a 96-well plate. (**a**) Screenshot of the Kinetics window of the ImagingWin user interface. Different colored curves represent the changes in algal photosynthetic efficiency Y(II) over time under different concentrations of Diuron treatment. For example, the black curve represents the control group, while other colored curves represent increasing Diuron concentrations (such as 0.3, 1, 3, 10, 30 nM, etc.). (**b**) Screenshot of the Image window of the ImagingWin user surface. (**c**) Concentration–response graphs of fluorescence parameters F and F′m and of the calculated Y(II) [64]. Copyright 2007 Biosensors and Bioelectronics.

Figure 4 illustrates the Diuron-induced fluorescence variations in a *Plasmodium keratinum* suspension within a 96-well plate. Experiments confirmed that this novel system enables exceptionally rapid and accurate phytotoxicity measurements using pulse amplitude modulation (PAM) fluorometry.

Baumann et al. [65] used PAM chlorophyll fluorometry to evaluate the toxicity of Cu, Cr, Zn, Cd, and Pb on seven marine macroalgae, correlating metal concentration with fluorescence yield as a toxicity indicator. They emphasized that chlorophyll fluorescence offers a sensitive alternative to microalgal bioassays for metal toxicity, with sensitivity comparable to other test organisms. Separately, Sjollema [66] investigated Diuron, Isoproturon, and Irgarol effects on *Selenastrum capricornutum* and *Dunaliella tertiolecta*, demonstrating a consistent correlation between algal growth inhibition and PSII inhibition. The study also assessed the impact of test conditions on toxicity results and validated PAM fluorescence against conventional ^14^C incorporation assays for accuracy.

### 3.4. Spectroscopy Hyphenated Techniques

Beyond these optical methods, techniques such as ultraviolet spectrophotometry [67,68], Raman spectrometry [69,70], and graphite furnace atomic absorption spectrometry [71,72] are also employed to evaluate toxicity in aquatic microorganisms and investigate underlying mechanisms. Biospectroscopy delivers comprehensive biochemical profiles from biological tissues and cells, allowing precise detection of molecular changes in composition or conformation. These techniques are valued for being rapid, non-destructive, objective, and reproducible. To overcome the limitations of individual methods, combined spectroscopic strategies have been developed. For example, Liu et al. [73] introduced a line-scanning hyperspectral fluorescence imaging (HFI) technique to decode quantum dot-encoded microbeads. Comparisons with flow cytometry confirmed the superior spectral resolution and multiplexing capacity of HFI. The system effectively decoded ssDNA-conjugated multicolored beads, demonstrating strong potential for biological and medical applications.

Electrogenerated chemiluminescence (ECL) integrates electrochemistry and chemiluminescence [74,75]. It involves applying an electric potential to a system containing a luminescent species, triggering redox reactions that generate excited-state intermediates. These intermediates emit light upon returning to the ground state. The emitted signal, captured using photomultiplier tubes or related optical devices, allows for spectral and intensity correlation with the analyte for highly sensitive detection. ECL provides notable advantages including low background noise, high signal-to-noise ratio, broad detection range, and ease of automation [76,77]. For instance, Guo et al. [78] constructed an ultrasensitive ECL biosensor using a γ-polyglutamic acid-graphene-luminol composite (γ-PGA-G-luminol) for mercury ion (Hg^2+^) detection. The ECL intensity correlated logarithmically with Hg^2+^ concentrations from 0.01 to 100 nmol/L (approx. 0.002–20 ppb; R^2^ = 0.99). The biosensor exhibited high selectivity toward Hg^2+^ with minimal interference from coexisting metal ions. This method offers a simple, rapid, cost-effective, and highly sensitive approach for Hg^2+^ analysis in aquatic environments.

## 4. Instrumental Analytical Methods II: Mass Spectrometric Techniques

The molecular fingerprints obtained via spectroscopy are crucial for hypothesis generation; however, definitive identification, precise quantification, and spatial mapping of toxicants require the unparalleled specificity and sensitivity of mass spectrometry (MS). This section examines the pivotal role of MS-based techniques, including elemental analysis by ICP-MS, organic pollutant profiling by GC/LC-MS, and spatial metabolomics via imaging MS, in advancing aquatic toxicology.

While spectroscopy is primarily used for qualitative identification of functional groups and species in a sample, mass spectrometry (MS) offers both qualitative and quantitative capabilities. As a result, MS has been widely applied in toxicity studies of aquatic microorganisms. It enables precise molecular weight quantification, formula assignment, and structural elucidation. Frequently coupled with separation techniques, MS extends its utility to a wide range of analytical scenarios. Furthermore, imaging mass spectrometry has emerged as a powerful extension, providing not only spatial localization of molecules but also visual distribution of their mass signals.

### 4.1. ICP-MS

Inductively coupled plasma mass spectrometry (ICP-MS) is a highly sensitive elemental analysis technique. It serves as a critical detector for hyphenated separation technologies and provides robust quantitative capabilities, including single-particle analysis. It is frequently coupled with methods such as HPLC for enhanced analytical performance.

#### 4.1.1. HPLC-ICP-MS

As a detector coupled with HPLC, ICP-MS enables tracking of isotopic signals across different elemental forms, simplifying chromatographic profiles and facilitating speciation analysis and quantification. HPLC-ICP-MS thus plays a crucial role in environmental monitoring and toxicological studies. Owing to its wide linear range and low detection limits, ICP-MS is particularly suitable for trace gold analysis. Soto-Alvaredo et al. [79] employed HPLC-ICP-MS to analyze tissues from Wistar rats following intraperitoneal injection of 10 nm Au NPs. By optimizing an enzymatic digestion protocol with proteinase K, the method achieved recovery rates of approximately 91% and successfully distinguished Au NPs from low-molecular-weight Au species. This approach enabled the detection of significant proportions (around 30%) of low-molecular Au species in liver and spleen samples, indicating that degradation processes play a pivotal role during the transport and accumulation of intraperitoneally injected Au NPs. While toxicity studies of Au(III) and Au NPs toward aquatic prokaryotes remain limited, Julita et al. [80] developed a reliable HPLC-ICP-MS method for quantifying both forms in algal matrices with high sensitivity, precision, and accuracy. Subsequent work demonstrated its utility in tracing the transformation and uptake of Au(III) and Au NPs in surface waters and algal cells, further elucidating their toxic effects on aquatic prokaryotes.

#### 4.1.2. SC-ICP-MS

**Figure 5:**
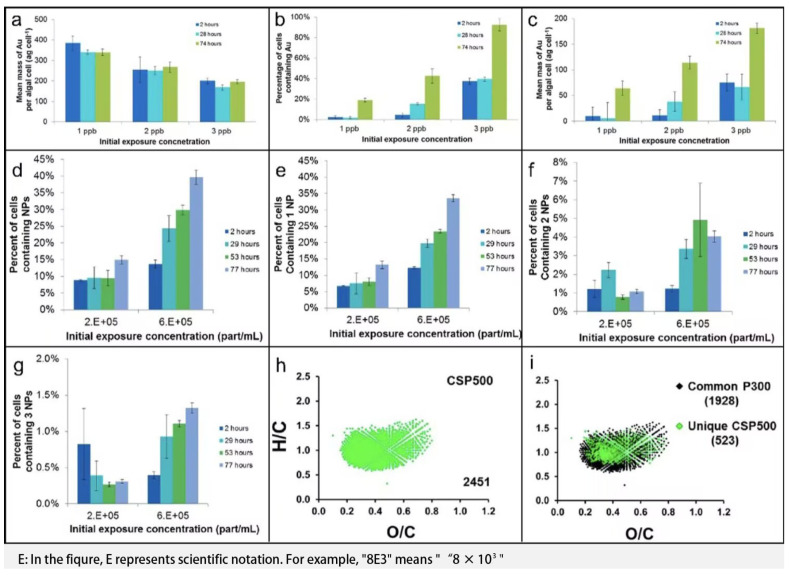
Information from acceptance tests for dissolved gold. (**a**) Mean quantity of Au per cell (**b**) The percentage of cells containing Au and reckoning by hemocytometer (**c**) Calculated Au quantity per cell after averaging. Information from absorption tests for nanoparticulate gold. (**d**) The proportion of all cells containing NPs, (**e**) Proportion of cells containing a single NP, (**f**) Proportion of cells containing 2 NPs/cell and (**g**) Proportion of cells containing 3 NPs/cell for 1:1 and 1:3 cell: NP exposure ratios throughout 77 h. Reproduced with permission [81]. Copyright 2018, ACS. Van Krevelen plot (**h**) shows the molecular formulas assigned to the original CSP500 sample (green). Van Krevelen plot (**i**) reveals 1928 common molecular formulas (black) designated in both pinewood biochar WSOC from 300 °C and CSP500, while 523 unique molecular formulas are designated to CSP500. Reproduced with permission [83]. Copyright 2016, ACS.

Traditional methods for quantifying cellular metal elements often rely on measuring total metal content to estimate average cellular concentrations. However, this approach is not only laborious and prone to contamination, but also limited by its inability to analyze individual cells. To overcome these constraints, Merrifield et al. [81] introduced single-cell inductively coupled plasma mass spectrometry (SC-ICP-MS), which enables precise quantification of metal concentrations in individual cells. Using SC-ICP-MS combined with cell counting strategies, they investigated the bioabsorption and adsorption of dissolved Au and Au nanoparticles in freshwater algae, as illustrated in Figure 5a–g. Similarly, Shen et al. [82] applied SC-ICP-MS to quantitatively assess copper uptake in P. aeruginosa cells and monitor their physiological status.

### 4.2. GC/LC-MS

Chromatography coupled with mass spectrometry is pivotal in environmental analysis. For example, GC–MS with solid-phase microextraction allows for accurate quantification of selenium removal in solutions [84,85]. Smith et al. [83] performed a comprehensive investigation into the sources of biochar water-soluble organic compounds (WSOC) derived from different biomass materials (including cellulose and lignin) across a pyrolysis temperature range of 300–500 °C, and evaluated their potential toxicity to the freshwater cyanobacterium Synechococcus. As illustrated conceptually in Figure 5h,i, advanced mass spectrometry analyses revealed that toxic WSOC from low-temperature (<400 °C) pinewood-derived biochar were primarily attributed to mono-, di-, and tri-substituted phenolic compounds originating from lignin, whereas toxic WSOC from cellulose-derived biochar exhibited acidic, bio-oil-like characteristics. The study further indicated that the total WSOC extracted from biochar decreased significantly with increasing pyrolysis temperature. For pinewood biochar produced above 400 °C, WSOC contained minimal to non-detectable levels of phenolic compounds, decreasing from 664.5 ± 6.2 mg DOC/kg biochar (300 °C) to 75.39 ± 0.42 mg DOC/kg biochar (500 °C), with no observable toxicity to cyanobacterial growth. Additionally, the study highlighted that imperfect pyrolysis conditions, such as the presence of cold spots and insufficient evacuation of pyrolysis intermediates, could lead to the generation of significant amounts of toxic WSOC. This integrated analytical approach effectively traces the origins of toxic WSOC and evaluates the impact of biochar-derived compounds on aquatic microorganisms.

Online solid-phase extraction (SPE) coupled with liquid chromatography–mass spectrometry (LC–MS) and biosensors is increasingly applied in environmental pollutant analysis. LC–MS, particularly tandem mass spectrometry (LC–MS/MS), offers superior specificity and sensitivity, making it a preferred technique [86,87]. For instance, it enables precise quantification of carbamazepine in microorganisms, bivalves, and algae at trace levels [88]. Additionally, Dell’Aversano et al. [89] employed hydrophilic interaction liquid chromatography coupled with electrospray mass spectrometry (HILIC/MS) to analyze diverse cyanobacterial toxins, including cylindrospermopsins, saxitoxins, anatoxins, and microcystins.

Cyanobacteria, ubiquitous photosynthetic bacteria inhabiting diverse aquatic systems commonly comprise toxic genera such as Anabaena, Microcystis, and Nodularia, known producers of microcystins (MCs) and other cyanotoxins [90]. Establishing rapid, universal detection methods is critical for effective bloom management [91]. Teta et al. [92] utilized high-resolution LC–MS integrated with molecular networking to rapidly identify both known and novel MC variants. Furthermore, Andrew et al. [93] established a robust UHPLC–MS/MS protocol for sensitive and accurate quantification of MCs and nodularins (NOD) in complex matrices such as shellfish, algal supplements, water, and bloom material. With high recovery and sensitivity, this method provides a reliable early-warning capability for toxin outbreaks, particularly supporting aquaculture protection during freshwater cyanobacterial blooms.

### 4.3. Imaging Mass Spectrometry

Matrix-assisted laser desorption ionization (MALDI), secondary ion mass spectrometry (SIMS), and desorption electrospray ionization (DESI) are prominent ionization techniques in mass spectrometry imaging (MSI), enabling simultaneous mapping of molecular spatial distributions and mass information. In natural products research, the main IMS platforms include MALDI-TOF, SIMS-TOF, and DESI-ion trap systems. While all are mass spectrometry-based, each technique offers distinct and complementary capabilities, making them collectively invaluable for tackling diverse biological challenges.

MALDI-TOF imaging is currently the most widely used IMS technology. Originally developed by Franz Hillencamp and Michael Karas in 1985, it was first applied to imaging by Kaufmann’s laboratory in 1994. To date, MALDI-based methods remain the dominant approach in natural product research using IMS.

**Figure 6:**
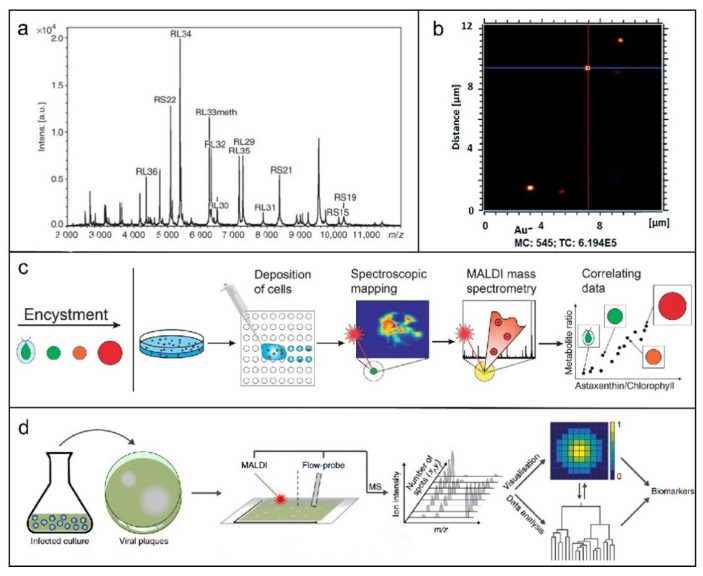
(**a**) A mass spectrum of an E. coli sample. Many of the mass peaks originate from proteins of ribosomal origin 20. Reproduced with permission [96]. Copyright 2009, Nature. (**b**) ToF-SIMS image of 200 nm Au NPs on an insulating sample carrier obtained in postponed extraction mode. Solid lines show the position for the determination of the lateral resolution in vertical (red) and horizontal (blue) directions [97]. Copyright 2019, The Royal Society of Chemistry. (**c**) The projected solo-cell method for joint examination of metabolites employs numerous analytical stages [98]. Copyright 2013, The Royal Society of Chemistry. (**d**) Indication of the workflow of ‘in plaque-MSI’ analysis [99]. Copyright 2019, Nature.

Matrix-assisted laser desorption/ionization time-of-flight mass spectrometry (MALDI-TOF-MS) is widely used to identify intact microorganisms and analyze microbial composition. Erhard et al. [94] employed it to characterize secondary metabolites—such as microcystins, micropeptides, and houttuynin—enabling discrimination between toxic and non-toxic algal blooms. Krader et al. [95] applied the technique to rapidly identify extremophilic prokaryotes, including archaea, sulfate-reducing bacteria, anaerobic phototrophs, and thermophiles. Freiwald et al. [96] demonstrated that MALDI-TOF-MS protein profiling facilitates precise bacterial classification, as exemplified by the reference spectrum of E. coli in Figure 6a. It also distinguishes pathogenic strains, such as Shiga toxin-producing and conventional Escherichia coli.

MALDI-TOF MS enables rapid identification of environmental bacteria, particularly aquatic pathogens such as Vibrio and Aeromonas species. It effectively discriminates between closely related pathogens (*Vibrio flexus* and *Vibrio freudii*) [100], and can identify up to 30 different Vibrio species directly from wastewater samples [101]. In a notable advance, Amanda et al. reported that fungal pigments can inhibit MALDI-TOF spectral quality, yet successfully applied the technique to fingerprint dark-pigmented fungi such as *Aspergillus niger*, marking a significant step forward in their identification [102]. Furthermore, Fagerer et al. [98] combined matrix-assisted laser desorption/ionization mass spectrometry with single-cell spectroscopy to profile metabolic features in freshwater algae (Figure 6c), offering a novel approach for assessing metabolic performance in algal biotechnology.

In general, MALDI-TOF MS is a cost-effective, rapid, and reliable technology for microbial identification, including anaerobic bacteria, fungi, archaea, and yeasts. Its early application in aquatic bacteriology supports three key areas: environmental monitoring, health management (e.g., diagnosis and treatment), and contaminant surveillance—particularly for emerging bacterial indicators of ecosystem disturbance [103]. The technique also facilitates early warning systems for detecting health impairments by analyzing microbiomes, such as those derived from crayfish tissues, based on spectral recognition scores [104].

SIMS has a narrower application scope than MALDI, focusing particularly on nanoparticle analysis. Originally conceptualized in 1910 and instrumentally developed by Liebl, Herzog, and Castaing around 1960, modern ultra-high-resolution SIMS can reveal biological processes—such as amino acid localization in Streptomyces or ammonia gradients in cyanobacterial heterocysts—and precisely determine nanoparticle positions in microalgae (Figure 6b) [97]. DESI, as a more recent ionization technique, remains less applied in aquatic microorganisms compared to MALDI and SIMS. Innovatively, Schleyer et al. [99] combined plaque assays with MSI to study metabolic transfer during infection, introducing “plaque-MSI” (Figure 6d).

## 5. Informatics Methods and Artificial Intelligence

The instrumental methods described above generate complex, high-dimensional data, and environmental exposures typically involve intricate mixtures of chemicals. To navigate this complexity, the field is increasingly turning to computational solutions. This section reviews how artificial intelligence and informatics methods are transforming toxicity assessment through predictive modeling, omics data integration, and large-scale environmental risk analysis.

The complexity of environmental mixtures and the vast datasets generated by modern omics and analytical technologies have necessitated a paradigm shift towards computational solutions. Artificial intelligence (AI), particularly machine learning (ML) and deep learning (DL), is rapidly transforming toxicity assessment by enabling predictive modeling, high-dimensional data integration, and automated pattern recognition. This section reviews how these informatics methods are being leveraged to predict toxicity for aquatic microorganisms, decipher mechanisms, and integrate complex data streams.

### 5.1. AI and Machine Learning for Predictive Toxicology

For predicting the toxicity of conventional chemicals, Zhang et al. [105] developed consensus AI models for the acute aquatic toxicity of chemicals towards Tetrahymena pyriformis, Daphnia magna, and Pimephales promelas (Fathead minnow). Their models achieved high prediction accuracies and identified critical molecular descriptors and structural alerts responsible for toxicity. Addressing the challenge of sparse data, Schlender et al. [106] systematically benchmarked state-of-the-art meta-learning techniques and demonstrated that multi-task Random Forest models robustly produced superior results for predicting aquatic toxicity across species in low-resource settings.

In the realm of nanomaterial (NM) toxicity prediction, several teams have made significant advances. Qi et al. [107] successfully developed quantitative nanostructure-activity relationship (nano-QSAR) classification and regression models to predict the oxidative stress potential induced by carbon nanomaterials and their binary mixtures in algal cells. Focusing on Daphnia magna immobilization, Balraadjsing et al. [108] showed that reliable classification models could be built from smaller datasets using algorithms like Random Forest and Artificial Neural Networks. Furthermore, Zhang et al. [109] constructed ML-based QSAR models to predict the combined toxicity of binary mixtures of metallic engineered nanoparticles, with their neural network-based model outperforming traditional component-based mixture models.

The prediction of mixture toxicity is an area where AI excels beyond conventional models. Wang et al. [110] proposed an Extended Generalized Concentration Addition (XGCA) model that incorporates a weighted molecular descriptor, proving more accurate in predicting the joint toxicity of antibiotic and nanoparticle mixtures to algae. In another study, Wang et al. [111] demonstrated that a Neural Network model, trained on the individual dose–response of components, more accurately predicted the combined toxicity of antibiotics to multiple aquatic species, even in the presence of dissolved organic matter. Qin et al. [112] applied 12 ML algorithms to predict the mixture toxicity of azole fungicide combinations, finding that a consensus model combining Support Vector Machine and Random Forest yielded the highest predictive accuracy. For high-throughput analysis, Duan et al. [113] developed a full strategy using a Random Forest algorithm trained on big-sample datasets generated via high-throughput printing technology. Lastly, Zhang et al. [114] employed an ensemble ML model to unravel the combined toxicity of micro(nano)plastics and environmental pollutants, complementing their findings with molecular dynamics simulations to elucidate the underlying mechanism.

### 5.2. Data Integration, Omics Informatics, and Macro-Scale Environmental Applications

AI is indispensable for interpreting high-dimensional “omics” data to link molecular perturbations with adverse outcomes. In omics data integration, Snyder et al. [115] used gas chromatography-mass spectrometry coupled with ML classifiers to analyze metabolomic profiles of tadpoles exposed to atrazine, successfully distinguishing exposed from control groups and revealing perturbations in key metabolic pathways. Chen et al. [116] combined metabolomics with ML methods to screen for serum biomarkers in workers occupationally exposed to TiO2 nanoparticles, identifying specific metabolites linked to exposure. As highlighted in the review by Li et al. [117], the integration of multi-omics technologies can holistically reveal molecular toxicity mechanisms, a process significantly enhanced by machine learning for data fusion and analysis.

For macro-scale environmental risk assessment and monitoring, AI enables powerful spatial and diagnostic tools. Chen et al. [118] utilized ML to generate high-resolution, spatially explicit global maps of pesticide mixture hazards in surface waters, identifying agricultural activities as the primary driver and defining optimal application intervals for mitigation. Nguyen et al. [119] developed an AI-powered water quality assessment (AiWA) approach using the XGBoost algorithm to rapidly predict the ecotoxicity of industrial wastewater effluents based on key physicochemical parameters. For forecasting ecological events, Huang et al. [120] created “Algae-Net,” a neural network model that accurately predicts algal density and species co-occurrence based on environmental drivers like total nitrogen and temperature, providing a robust tool for early warning of harmful algal blooms. The integration of AI with sensor technology is also advancing, as reviewed by Sree et al. [121], who noted that wireless and AI technologies are enabling real-time, intelligent environmental monitoring through next-generation biosensing platforms.

### 5.3. Challenges and Future Perspectives

Despite remarkable progress, the field faces persistent challenges. Data quality and availability remain a primary bottleneck. An earlier review by Chen et al. [122] on nano-QSARs concluded that a lack of consistent, well-characterized experimental data severely hindered model development and validation. Model interpretability and trust are critical for regulatory acceptance. Efforts like those by Huang et al. [120], who used gradient-based attribution methods to identify key environmental drivers in their algal bloom model, are crucial for enhancing transparency. Generalization in low-resource scenarios requires innovative approaches, such as the meta-learning strategies benchmarked by Schlender et al. [106].

The future lies in intelligent, integrated strategies. This involves the convergence of AI with high-throughput experimental platforms, mechanistic modeling like molecular dynamics simulations, and real-time data streams from environmental sensor networks. The ultimate goal is the development of dynamic, predictive, next-generation risk assessment frameworks that transition from reactive hazard identification to proactive system protection.

## 6. Nanobiochips

Complementing the data-intensive and laboratory-centric approaches previously discussed, a parallel trend focuses on miniaturization and automation for rapid, on-site screening. Nanobiochips, which integrate biological sensing elements with microfluidic platforms, embody this trend. The final methodological section evaluates the potential of these devices to provide portable, real-time toxicity monitoring in aquatic environments.

Biochip technology, originating from nucleic acid hybridization [123], typically refers to high-density microarrays comprising biological molecules (e.g., DNA, peptides, proteins, or glycans) immobilized in predefined patterns on a solid substrate. Among later developments are microfluidic and liquid-phase biochips. Microfluidic chips integrate sample preparation, reaction, separation, and detection into a miniaturized system, enabling automated analytical workflows with applications in biomedicine, chemistry, and clinical diagnostics. Liquid-phase biochips are often used in immunoassays, nucleic acid analysis, enzyme activity tests, and receptor-ligand studies. While microarray chips allow high-throughput screening of multiple genes, their limitations have motivated the use of diversified chip technologies in detecting aquatic microorganism toxicity. Despite their sensitivity and accuracy, conventional methods are often restricted to specialized laboratories and require bulky, sophisticated equipment as well as highly trained operators. Biochip technology offers a promising alternative with improvements in speed, automation, reproducibility, and cost-effectiveness, alongside portability. Algae are widely used in toxicity bioassays on account of their high sensitivity to environmental stressors, even at trace levels of pollutants [124,125]. Since many contaminants affect photosynthetic activity, its inhibition serves as a rapid and sensitive indicator of toxicity. Algae-based biosensors have thus been shown to detect trace pollutants effectively and are suitable for simple, low-cost, on-site preliminary screening.

**Figure 7:**
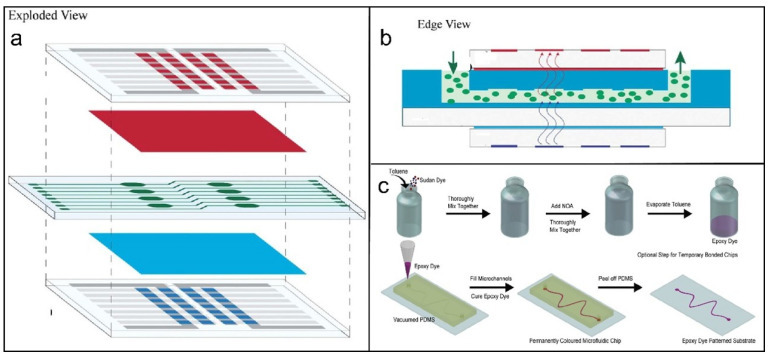
(**a**) Exploded view and (**b**) edge view of the schematic of the proposed fluorescence sensor. Reproduced with authorization [126]. Copyright 2012, Royal Society of Chemistry. (**c**) Epoxy dye preparation and maintaining the microfluidic chip process [128]. Copyright 2022, Springer.

In 2009, Chen et al. developed a multiplex PCR-gene chip platform for simultaneous detection of three major foodborne pathogens—*Shigella* spp., *Salmonella enterica*, and enterohemorrhagic *E. coli* O157 [100]. Although seven bacterial types were tested, only these three showed positive amplification, with a detection sensitivity of approximately 8 pg for both genomic DNA and pure cultures. Results from food samples correlated well with conventional bacteriological cultures, confirming the method’s high specificity and sensitivity for pathogen screening. Florent et al. developed a pioneering algae-based micro-fluorescence sensor by integrating organic light-emitting diodes (OLEDs) and carbon-based photodetectors on a microfluidic chip platform [126]. A blue OLED served as the excitation source, while a blended organic photodetector captured emitted signals. Acid/base dyes and metal complex filters were assembled with carbon-based optoelectronic components to form a complete fluorescence detection system, which was embedded in a PDMS microfluidic chip. This sensor detected herbicide toxicity—e.g., Diuron on *Chlamydomonas reinhardtii*—via chlorophyll fluorescence inhibition at concentrations as low as 11 nM. Offering high sensitivity and portability, this organic bioassay meets growing demands for on-site water toxicity monitoring using microfluidic biosensors. The chip design is illustrated in Figure 7b; a similar structure is shown in Figure 7c [127,128]. Chip-based platforms represent a modern molecular biology tool valued for rapid, accurate, and high-throughput automation. They are applied in diverse biological fields such as bacterial epidemiology, gene identification, mutation analysis, DNA sequencing, and expression profiling [129]. Microfluidic chips enable heavy metal toxicity assessment by analyzing microalgal motility [126]. These systems typically integrate microculture units that support direct dose–response studies [130]. Transient immobilization of microalgae in chemically stable microfluidic chambers enables sensitive toxicity bioassays without compromising cellular viability. While initially applied for heavy metal detection, this method holds promise for screening a wider range of contaminants. With demonstrated efficacy against other toxins, it may emerge as an efficient and practical alternative to conventional laboratory-based toxicity assays.

## 7. Other Technologies

Beyond conventional methods such as mass spectrometry, and chips, emerging methodology including microscopy, flow cytometry, artificial intelligence, and integrated technological platforms have been increasingly being adopted by researchers.

### 7.1. Microscopy

Optical microscopy identifies chromosomal mutations, nuclear abnormalities, and histopathological changes induced by chemical pollutants. Fluorescence microscopy detects genotoxicity or mutagenicity by revealing DNA damage, while electron microscopy reveals ultrastructural alterations in cells. Collectively, these techniques provide multifaceted insights into the toxicity of chemicals toward aquatic microorganisms. As early as 1993, Liao et al. [130] conducted a comprehensive safety assessment of the Cistanche tubulosa health product Memoregain®. Their in vitro Ames test using five Salmonella typhimurium strains showed no signs of increased reverse mutation up to 5 mg/plate, and exposure did not increase the frequency of chromosomal aberrations in CHO-K1 cells. In vivo micronucleus tests in ICR mice and a 28-day repeated oral dose toxicity study in rats further confirmed the absence of observable adverse effects, supporting the product’s safety for human consumption. Beyond such toxicological screening, advanced microscopy techniques offer direct morphological and ultrastructural evidence. Nancharaiah et al. [131] introduced a confocal laser scanning microscopy (CLSM)-based approach for assessing microalgal ecotoxicity at single-cell resolution. More recently, Ji et al. [132] applied SEM to evaluate the effects of ZnO nanoparticles on Chlorella. Additionally, Roose-Amsaleg et al. [133] utilized interferometric light microscopy to quantify viral abundance in riverine environments.

### 7.2. Flow Cytometry

Flow cytometry has emerged through interdisciplinary integration of cell biology, biotechnology, laser optics, fluorescence chemistry, hydrodynamics, and computer science. Flow cytometry probes cellular characteristics (i.e., size, granularity, and antigen expression) by detecting scattered and fluorescent light signals from laser-interrogated cells in flow, allowing precise assessment of aquatic microorganisms. Harry et al. [134] applied flow cytometry to evaluate the impact of treated wastewater on stream microbial communities. Props et al. [135] developed a novel approach for detecting microbial contamination in drinking water, combining real-time flow cytometry (RT-FCM) with advanced fingerprinting analysis to characterize both natural communities and individual species.

### 7.3. Multimodal Integrated Approach

The study of complex biological systems increasingly requires the integration of multiple analytical technologies to obtain comprehensive insights. In aquatic toxicology, many researchers are transitioning from single-method approaches to combined-technique strategies. For instance, Sekine et al. [136] employed dark-field optical microscopy, high-resolution electron microscopy, and nanoscale secondary ion mass spectrometry (NanoSIMS) to investigate interactions between silver nanoparticles (Ag-NPs) and green algae. This multi-modal approach provided complementary strengths in fidelity, spatial resolution, and elemental identification, revealing that Ag-NP toxicity depends primarily on particle size and surface properties. Tian et al. [137] introduced a novel hybrid technique combining single-probe mass spectrometry and fluorescence microscopy for correlative imaging, overcoming the drawbacks of MALDI-MSI to enable spatial mapping of metabolites and proteins in amyloid plaques. This fusion significantly enhanced spatial resolution, enabling precise visualization of fine structural details and metabolite biomarkers associated with amyloid pathology.

## 8. Synthesis of Methodological Advantages and Limitations

A critical understanding of the strengths and constraints inherent to each methodological category is essential for selecting the most appropriate tool for a given research or monitoring objective. The following sections synthesize the principal advantages and limitations of the major techniques discussed in this review, moving from direct biological assays to advanced instrumental and computational approaches.

### 8.1. General Toxicity Assays (Biological and Chemical Methods)

The conventional bioassays and chemical methods described in Section 2 offer a foundational approach to toxicity screening. Their primary advantages stem from direct ecological relevance, cost-effectiveness, potential for high throughput, and the availability of well-standardized protocols (e.g., ISO, OECD). Certain formats, particularly some luminescent bacteria tests, have been adapted for field-portable use, enabling rapid on-site screening. However, these methods are characterized by several limitations: they typically provide limited mechanistic insight into toxic action; results can be variable and highly dependent on specific test conditions (e.g., pH, temperature); they exhibit low specificity for identifying individual contaminants within complex mixtures; they are generally less sensitive to chronic, sub-lethal effects compared to molecular endpoints; and they require the maintenance of live test organism cultures, which can be logistically demanding.

### 8.2. Spectroscopic Techniques

Spectroscopic methods (Section 3), including FTIR, hyperspectral imaging, and fluorescence spectroscopy, provide a non-destructive window into the biochemical states of aquatic microorganisms. Key advantages include their non-destructive nature, ability to provide molecular “fingerprinting,” minimal sample preparation requirements, and, for techniques like HSI, the capability for spatial imaging. Methods such as chlorophyll fluorescence imaging can be rapid for high-throughput screening. The main limitations of these techniques are their generally lower sensitivity compared to mass spectrometry, the generation of complex data that often requires advanced chemometric analysis for interpretation, limited applicability for compounds without distinct chromophores or vibrational signatures, and the potential for spectral overlap in complex environmental matrices which can complicate analysis.

### 8.3. Mass Spectrometric Techniques

Mass spectrometry-based methods (Section 4) represent the gold standard for definitive identification, precise quantification, and spatial mapping of toxicants. Their advantages are unparalleled: high sensitivity and specificity, excellent quantitative capabilities, the ability to perform speciation analysis (e.g., via ICP-MS), provision of detailed structural information, and spatial mapping of molecules through imaging MS (e.g., MALDI, SIMS). Conversely, these capabilities come with significant limitations: high instrument acquisition and operational costs, often complex and time-consuming sample preparation protocols, typically destructive analysis (precluding sample re-use), a requirement for highly skilled operators, and data interpretation that can be challenging and requires expert knowledge.

### 8.4. Informatics and Artificial Intelligence Approaches

Computational methods (Section 5) are transforming toxicity assessment by handling complex, high-dimensional data. Their core advantages include powerful predictive capability for toxicity (e.g., via QSAR/nano-QSAR models), the ability to integrate and model high-dimensional omics data, the capacity to predict mixture toxicity beyond traditional models, and the enabling of large-scale environmental risk mapping and forecasting (e.g., for algal blooms or pesticide hazards). The primary limitations of AI/ML approaches are their high dependency on the quality, quantity, and consistency of input data; the frequent “black box” nature of models which can hinder interpretability and regulatory acceptance; the risk of overfitting to training datasets; and the need for cross-disciplinary expertise for proper model development, validation, and application.

### 8.5. Nanobiochips and Integrated Sensor Platforms

Nanobiochip technology (Section 6) aims to miniaturize and automate toxicity screening. Its notable advantages are low consumption of samples and reagents, high potential for automation and throughput, portability for on-site deployment, and the capacity for real-time or near-real-time monitoring. The limitations currently impeding wider adoption include complex and often costly device fabrication processes, stability issues of integrated biological sensing elements (e.g., enzymes, whole cells), susceptibility to clogging when analyzing complex environmental samples, limited multiplexing capability in fully integrated portable devices, and performance that can be sensitive to sample matrix effects.

## 9. Comparative Summary of Methodologies

The preceding sections have detailed a diverse suite of methods for assessing chemical toxicity to aquatic microorganisms. To facilitate informed selection and highlight strategic trade-offs, Table 2 provides a consolidated comparison based on key operational and performance parameters: sensitivity (or limit of detection), approximate cost, throughput, principal advantages, and major limitations. This synthesis is not merely a tabulation but a framework for critical evaluation, underscoring that no single method is universally superior; the optimal choice is dictated by the specific research question, required information depth, and practical constraints.

A critical examination of Table 2 reveals fundamental methodological trade-offs that guide their application. Biological assays (e.g., luminescent bacteria) excel in ecological relevance and screening throughput at low cost but sacrifice mechanistic specificity and can be confounded by complex sample matrices. Spectroscopic techniques offer non-invasive, molecular-level insights but generally possess lower sensitivity and generate data requiring advanced analysis. Mass spectrometry stands as the benchmark for sensitivity, specificity, and definitive identification, yet this comes with high costs, operational complexity, and destructive analysis. Nanobiochips promise the future of decentralized, real-time monitoring but currently face challenges in robustness, multiplexing, and handling real-world environmental samples. Informatics approaches revolutionize predictive capacity and data integration but are fundamentally constrained by data quality and face hurdles in model transparency and validation.

Consequently, the trend in advanced aquatic toxicology is not towards reliance on a single method, but towards tiered and integrated strategies. A common paradigm involves using high-throughput, cost-effective bioassays or biosensors for initial screening and monitoring. Compounds or samples flagged as toxic are then subjected to targeted instrumental analysis (e.g., LC-MS/MS, ICP-MS) for precise quantification and identification. Informatics tools can guide this process by predicting toxicity, prioritizing contaminants, and integrating heterogeneous data from multiple sources to construct adverse outcome pathways. This synergistic, multi-methodological approach leverages the respective strengths of each technique while mitigating their individual limitations, ultimately leading to a more comprehensive and mechanistic understanding of chemical impacts on aquatic microorganisms.

## 10. Conclusions

This review provides a systematic overview of methodological advances in assessing the toxicity of chemicals toward aquatic microorganisms, covering biological, chemical, instrumental, and informatics-based approaches. Biological assays offer direct insight into the integrated toxic effects on ecosystems, with benefits such as low cost and operational simplicity; however, they often lack ecological relevance and are unable to quantify specific pollutants. Conventional chemical methods support both qualitative and quantitative analysis but are generally outperformed in accuracy and precision by modern instrumental techniques. The latter deliver high precision, rapid processing, and superior reproducibility, enabling deeper mechanistic understanding of toxicity. Informatics methods open new pathways for evaluating complex toxicological systems through computational and data-driven strategies. We hope this review will inspire continued development of novel detection methodologies in the field.

## Figures and Tables

**Table 1:** Overview of common standardized bioassays for toxicity assessment in aquatic microorganisms.

Method	Standard Organization/Number	Test Organism	Endpoint	Typical Application
Luminescent bacteria inhibition test	ISO [14]/11348	Vibrio fischeri	Inhibition of bioluminescence	Acute toxicity screening of water and wastewater
Algal growth inhibition test	OECD [15]/201, ISO [16]/8692	Freshwater algae (e.g., Pseudokirchneriella subcapitata)	Growth rate inhibition	Toxicity of chemicals, effluents
Nitrification inhibition test	ISO [17]/9509	Activated sludge nitrifying bacteria	Ammonia oxidation rate	Toxicity to wastewater treatment processes
Daphnia acute immobilization test	OECD [18]/202	Daphnia magna	Immobilization	Acute toxicity of chemicals
Fish acute toxicity test	OECD [19]/203	Fish (e.g., Oncorhynchus mykiss)	Mortality	Acute toxicity of chemicals

**Table 2:** Comparative summary of methodologies for assessing toxicity to aquatic microorganisms.

Method	Sensitivity/LOD	Cost	Throughput	Key Advantages	Limitations
Luminescent bacteria assay	Moderate (µg/L)	Low	High	Rapid, cost-effective, field-portable	Limited to specific toxins, matrix interference
FTIR	Low (mg/L)	Moderate	Moderate	Non-destructive, molecular fingerprinting	Low sensitivity, requires spectral interpretation
HPLC-ICP-MS	High (ng/L)	High	Moderate	Speciation analysis, high precision	Expensive, skilled operator needed
Nanobiochip	High (pg/mL)	Moderate	High	Portable, real-time detection	Limited multiplexing, stability issues
AI/QSAR	N/A (predictive)	Low	Very High	High-throughput, predictive capability	Data dependency, model validation needed

## Data Availability

No new data were created or analyzed in this study. Data sharing is not applicable to this article.
